# Metagenomics of urban sewage identifies an extensively shared antibiotic resistome in China

**DOI:** 10.1186/s40168-017-0298-y

**Published:** 2017-07-19

**Authors:** Jian-Qiang Su, Xin-Li An, Bing Li, Qing-Lin Chen, Michael R. Gillings, Hong Chen, Tong Zhang, Yong-Guan Zhu

**Affiliations:** 1 Key Lab of Urban Environment and Health, Institute of Urban Environment, Chinese Academy of Sciences, 1799 Jimei Road, 361021 Xiamen, China; 2 University of Chinese Academy of Sciences, 19A Yuquan Road, 100049 Beijing, China; 3 State Key Laboratory of Urban and Regional Ecology, Research Center for Eco-Environmental Sciences, Chinese Academy of Sciences, 100085 Beijing, China; 4 Environmental Biotechnology Laboratory, The University of Hong Kong, Pokfulam Road, Hong Kong, China; 5 Division of Energy & Environment, Graduate School at Shenzhen, Tsinghua University, 518055 Shenzhen, China; 6 Department of Biological Sciences, Macquarie University, Sydney, NSW 2109 Australia; 7 Department of Environmental Engineering, College of Environmental and Resource Sciences, Zhejiang University, Hangzhou, 310058 China

**Keywords:** Antibiotic resistance, Environment, Pollution, Evolution, Human gut microbiome

## Abstract

**Background:**

Antibiotic-resistant pathogens are challenging treatment of infections worldwide. Urban sewage is potentially a major conduit for dissemination of antibiotic resistance genes into various environmental compartments. However, the diversity and abundance of such genes in wastewater are not well known.

**Methods:**

Here, seasonal and geographical distributions of antibiotic resistance genes and their host bacterial communities from Chinese urban sewage were characterized, using metagenomic analyses and 16S rRNA gene-based Illumina sequencing, respectively.

**Results:**

In total, 381 different resistance genes were detected, and these genes were extensively shared across China, with no geographical clustering. Seasonal variation in abundance of resistance genes was observed, with average concentrations of 3.27 × 10^11^ and 1.79 × 10^12^ copies/L in summer and winter, respectively. Bacterial communities did not exhibit geographical clusters, but did show a significant distance-decay relationship (*P* < 0.01). The core, shared resistome accounted for 57.7% of the total resistance genes, and was significantly associated with the core microbial community (*P* < 0.01). The core human gut microbiota was also strongly associated with the shared resistome, demonstrating the potential contribution of human gut microbiota to the dissemination of resistance elements via sewage disposal.

**Conclusions:**

This study provides a baseline for investigating environmental dissemination of resistance elements and raises the possibility of using the abundance of resistance genes in sewage as a tool for antibiotic stewardship.

**Electronic supplementary material:**

The online version of this article (doi:10.1186/s40168-017-0298-y) contains supplementary material, which is available to authorized users.

## Background

Antibiotic resistance is one of the most serious global threats to human health, challenging the treatment of life-threatening infections [1]. The widespread use of antibiotics in humans and animals is the main selective driving force of the emergence and dissemination of antibiotic resistance, and thus the cure is also the cause [2, 3]. Antibiotic resistant pathogens now occur at high frequencies in clinical contexts, and are increasingly being found in environmental settings, such as water bodies [4], soils [5], and animal feces [6]. In particular, the frequent presence of multi-antibiotic resistant “superbugs” in human feces predicts a return to the pre-antibiotic era, where a growing number of infections can no longer be treated using the current arsenal of drugs [7, 8].

Consequently, the World Health Organization has endorsed a Global Action Plan on Antimicrobial Resistance, which calls upon all nations to adopt mitigation strategies within 2 years [9]. However, there is still more to understand about the ecology and evolution of antibiotic resistance. In particular, not enough is known about the properties of the microbial resistome in ecosystems dominated by humans, and how to monitor such environments in order to evaluate their potential for promoting the evolution of antibiotic resistance.

Municipal wastewater treatment plants (WWTPs) receive and digest millions of tons of domestic sewage. Adults harbor significant quantities of resistance genes in their gut microbiome [10], and consequently WWTPs, especially influents are likely to be a critical hub for the evolution and spread of anthropogenically derived resistance genes into natural environments [11, 12]. In China, more than 3700 municipal WWTPs have been constructed to treat urban sewage, with a combined capacity of 157 billion liters per day [13]. In each of these facilities, sewage from tens to hundreds of thousands of individuals creates an enormous biological reactor where bacteria, and resistance genes are exposed to significant concentrations of selective agents such as antimicrobial agents, disinfectants and heavy metals [14]. The selective pressures exerted by these agents, together with the presence of dense bacterial populations facilitates selection of antibiotic resistance and the generation of additional resistant bacteria via horizontal gene transfer (HGT) [15]. This makes sewage a vast repository of bacteria that carry and exchange resistance genes. In this respect, resistance genes detected in sewage might represent the resistance burden of their urban populations. Resistance profiles in sewage could then reflect the structure and diversity of resistant bacteria in the gastrointestinal tracts of urban residents within the WWTP catchment. This may be especially true when the WWTP mainly treats domestic wastewater without significant contributions from agricultural and industrial sources [16]. A nation-wide survey of resistance elements in sewage (untreated influent) could then provide a rapid and efficient method for assessing the burden of antibiotic resistance from urban populations.

Urban sewage compositions are subject to strong temporal and environmental variation in conditions. However, if and how the composition of microbial community and antibiotic resistomes change with seasons and regions in urban sewage have not been extensively investigated. To address this need, 116 urban sewage samples were collected from 32 WWTPs in 17 major Chinese cities during summer and winter. Sampling sites were specifically chosen to reflect diverse climatic conditions, economic development levels and urban geography. By combining metagenomic analyses and Illumina sequencing of 16S rRNA genes, the seasonal and geographical variations of antibiotic resistome and corresponding microbial community structure were characterized.

## Methods

### Sample collection and DNA extraction

A total of 116 sewage samples were collected from 32 WWTPs influents in 17 major Chinese cities during August 2014 (summer, *n* = 59) and February 2014 (winter, *n* = 57). All the untreated influent samples from each WWTP were taken within two consecutive days without recent rainfall to exclude the effect of the weather. Detailed information on these samples is summarized in Table 1 and Additional file 1: Table S1. All sewage samples from WWTPs were collected in 400-mL sterilized containers and were mixed with 100% ethanol at a volume ratio of 1:1 for biomass fixation. The fixed samples were kept on ice and were immediately delivered to laboratory. The microbial cells from 400 mL of fixed sample were pelleted by centrifuging at 9500 g for 20 min at 4 °C. All pellets were stored at −20 °C before DNA extraction.

**Table 1:** Information of WWTP sewage samples

Sample name^a^	Name of city	Name of city (abbr.)	Name of WWTP	Code of WWTP	Administrative regions	Treatment technology
S_BJ_QH_1	Beijing	BJ	Qinghe	QH	Beijing	Anaerobic-Anoxic-Oxic (A2/O)
S_BJ_QH_2	Beijing	BJ	Qinghe	QH	Beijing	Anaerobic-Anoxic-Oxic (A2/O)
S_BJ_XHM_1	Beijing	BJ	Xiaohongmen	XHM	Beijing	Anaerobic-Anoxic-Oxic (A2/O)
S_BJ_XHM_2	Beijing	BJ	Xiaohongmen	XHM	Beijing	Anaerobic-Anoxic-Oxic (A2/O)
S_CQ_JGS_1	Chongqing	CQ	Jiguanshi	JGS	Chongqing	Anaerobic-Anoxic-Oxic (A2/O)
S_CQ_JGS_2	Chongqing	CQ	Jiguanshi	JGS	Chongqing	Anaerobic-Anoxic-Oxic (A2/O)
S_CQ_TJT_1	Chongqing	CQ	Tangjiatuo	TJT	Chongqing	Anaerobic-Anoxic-Oxic (A2/O)
S_CQ_TJT_2	Chongqing	CQ	Tangjiatuo	TJT	Chongqing	Anaerobic-Anoxic-Oxic (A2/O)
S_GZ_KFQ_1	Guangzhou	GZ	Kaifaqu	KFQ	Guangdong	Oxidation ditch
S_GZ_KFQ_2	Guangzhou	GZ	Kaifaqu	KFQ	Guangdong	Oxidation ditch
S_GZ_LD_1	Guangzhou	GZ	Liede	LD	Guangdong	Anaerobic-Anoxic-Oxic (A2/O)
S_GZ_LD_2	Guangzhou	GZ	Liede	LD	Guangdong	Anaerobic-Anoxic-Oxic (A2/O)
S_HK_SHX_1	Hong Kong	HK	Shihuxu	SHX	Hong Kong	Anaerobic-Anoxic-Oxic (A2/O)
S_HK_SHX_2	Hong Kong	HK	Shihuxu	SHX	Hong Kong	Anaerobic-Anoxic-Oxic (A2/O)
S_HK_ST_1	Hong Kong	HK	Shatin	ST	Hong Kong	Anaerobic-Anoxic-Oxic (A2/O)
S_HK_ST_2	Hong Kong	HK	Shatin	ST	Hong Kong	Anaerobic-Anoxic-Oxic (A2/O)
S_HZ_LA_1	Hangzhou	HZ	Lin’an	LA	Zhejiang	Oxidation ditch
S_HZ_LA_2	Hangzhou	HZ	Lin’an	LA	Zhejiang	Oxidation ditch
S_HZ_QG_1	Hangzhou	HZ	Qige	QG	Zhejiang	Anaerobic-Anoxic-Oxic (A2/O)
S_HZ_QG_2	Hangzhou	HZ	Qige	QG	Zhejiang	Anaerobic-Anoxic-Oxic (A2/O)
S_LS_1#_1	Lasa	LS	1#	1#	Tibet	Anaerobic-Anoxic-Oxic (A2/O)
S_LS_1#_2	Lasa	LS	1#	1#	Tibet	Anaerobic-Anoxic-Oxic (A2/O)
S_LS_2#_1	Lasa	LS	2#	2#	Tibet	Anaerobic-Anoxic-Oxic (A2/O)
S_LS_2#_2	Lasa	LS	2#	2#	Tibet	Anaerobic-Anoxic-Oxic (A2/O)
S_LY_1	Longyan	LY	Longyan	LY	Fujian	Anaerobic-Anoxic-Oxic (A2/O)
S_LY_2	Longyan	LY	Longyan	LY	Fujian	Anaerobic-Anoxic-Oxic (A2/O)
S_LZ_AN_1	Lanzhou	LZ	An’ning	AN	Gansu	Sequence Batch Reactor (SRB)
S_LZ_AN_2	Lanzhou	LZ	An’ning	AN	Gansu	Sequence Batch Reactor (SRB)
S_NJ_DC_1	Nanjing	NJ	Dachang	DC	Jiangsu	Anaerobic-Anoxic-Oxic (A2/O)
S_NJ_DC_2	Nanjing	NJ	Dachang	DC	Jiangsu	Anaerobic-Anoxic-Oxic (A2/O)
S_NJ_JXZ_1	Nanjing	NJ	Jiangxinzhou	JXZ	Jiangsu	Anaerobic-Anoxic-Oxic (A2/O)
S_NJ_JXZ_2	Nanjing	NJ	Jiangxinzhou	JXZ	Jiangsu	Anaerobic-Anoxic-Oxic (A2/O)
S_SH_MH_1	Shanghai	SH	Minghang	MH	Shanghai	Anaerobic-Anoxic-Oxic (A2/O)
S_SH_MH_2	Shanghai	SH	Minghang	MH	Shanghai	Anaerobic-Anoxic-Oxic (A2/O)
S_SH_MHSZ_1	Shanghai	SH	Minghangshuizhi	MHSZ	Shanghai	Anaerobic-Anoxic-Oxic (A2/O)
S_SH_MHSZ_2	Shanghai	SH	Minghangshuizhi	MHSZ	Shanghai	Anaerobic-Anoxic-Oxic (A2/O)
S_SZ_GM_1	Shenzhen	SZ	Guangming	GM	Guangdong	Anaerobic-Anoxic-Oxic (A2/O)
S_SZ_GM_2	Shenzhen	SZ	Guangming	GM	Guangdong	Anaerobic-Anoxic-Oxic (A2/O)
S_SZ_LF_1	Shenzhen	SZ	Luofang	LF	Guangdong	Oxidation ditch
S_SZ_LF_2	Shenzhen	SZ	Luofang	LF	Guangdong	Oxidation ditch
S_TJ_XYL_1	Tianjin	TJ	Xianyanglu	XYL	Tianjin	Anoxic/Oxic (A/O)
S_TJ_XYL_2	Tianjin	TJ	Xianyanglu	XYL	Tianjin	Anoxic/Oxic (A/O)
S_TJ_ZGZ_1	Tianjin	TJ	Zhangguizhuang	ZGZ	Tianjin	Anoxic/Oxic (A/O)
S_TJ_ZGZ_2	Tianjin	TJ	Zhangguizhuang	ZGZ	Tianjin	Anoxic/Oxic (A/O)
S_WH_NTZ_1	Wuhan	WH	Nantaizi	NTZ	Hubei	Carrousel oxidation ditch
S_WH_NTZ_2	Wuhan	WH	Nantaizi	NTZ	Hubei	Carrousel oxidation ditch
S_WH_TXH_1	Wuhan	WH	Tangxunhu	TXH	Hubei	DE oxidation ditch
S_WH_TXH_2	Wuhan	WH	Tangxunhu	TXH	Hubei	DE oxidation ditch
S_WLMQ_HD_1	Wulumuqi	WLMQ	Hedong	HD	Sinkiang	Sequence Batch Reactor (SRB)
S_WLMQ_HD_2	Wulumuqi	WLMQ	Hedong	HD	Sinkiang	Sequence Batch Reactor (SRB)
S_WLMQ_HX_1	Wulumuqi	WLMQ	Hexi	HX	Sinkiang	Sequence Batch Reactor (SRB)
S_WLMQ_HX_2	Wulumuqi	WLMQ	Hexi	HX	Sinkiang	Sequence Batch Reactor (SRB)
S_XA_SW_1	Xi'an	XA	Sanwu	SW	Shan’xi	Orbal oxidation ditch
S_XA_WW_1	Xi'an	XA	Wuwu	WW	Shan’xi	Orbal oxidation ditch
S_XA_WW_2	Xi'an	XA	Wuwu	WW	Shan’xi	Anaerobic-Anoxic-Oxic (A2/O)
S_XM_JM_1	Xiamen	XM	Jimei	JM	Fujian	Orbal oxidation ditch
S_XM_JM_2	Xiamen	XM	Jimei	JM	Fujian	Orbal oxidation ditch
S_XM_QP_1	Xiamen	XM	Qianpu	QP	Fujian	Oxidation ditch
S_XM_QP_2	Xiamen	XM	Qianpu	QP	Fujian	Oxidation ditch
W_BJ_QH_1	Beijing	BJ	Qinghe	QH	Beijing	Anaerobic-Anoxic-Oxic (A2/O)
W_BJ_QH_2	Beijing	BJ	Qinghe	QH	Beijing	Anaerobic-Anoxic-Oxic (A2/O)
W_BJ_XHM_1	Beijing	BJ	Xiaohongmen	XHM	Beijing	Anaerobic-Anoxic-Oxic (A2/O)
W_BJ_XHM_2	Beijing	BJ	Xiaohongmen	XHM	Beijing	Anaerobic-Anoxic-Oxic (A2/O)
W_CQ_JQS_1	Chongqing	CQ	Jiguanshi	JGS	Chongqing	Anaerobic-Anoxic-Oxic (A2/O)
W_CQ_JQS_2	Chongqing	CQ	Jiguanshi	JGS	Chongqing	Anaerobic-Anoxic-Oxic (A2/O)
W_CQ_TJT_1	Chongqing	CQ	Tangjiatuo	TJT	Chongqing	Anaerobic-Anoxic-Oxic (A2/O)
W_CQ_TJT_2	Chongqing	CQ	Tangjiatuo	TJT	Chongqing	Anaerobic-Anoxic-Oxic (A2/O)
W_GZ_LD_1	Guangzhou	GZ	Liede	LD	Guangdong	Anaerobic-Anoxic-Oxic (A2/O)
W_GZ_LD_2	Guangzhou	GZ	Liede	LD	Guangdong	Anaerobic-Anoxic-Oxic (A2/O)
W_HK_SHX_1	Hong Kong	HK	Shihuxu	SHX	Hong Kong	Anaerobic-Anoxic-Oxic (A2/O)
W_HK_SHX_2	Hong Kong	HK	Shihuxu	SHX	Hong Kong	Anaerobic-Anoxic-Oxic (A2/O)
W_HK_ST_1	Hong Kong	HK	Shatin	ST	Hong Kong	Anaerobic-Anoxic-Oxic (A2/O)
W_HK_ST_2	Hong Kong	HK	Shatin	ST	Hong Kong	Anaerobic-Anoxic-Oxic (A2/O)
W_HZ_LA_1	Hangzhou	HZ	Lin’an	LA	Zhejiang	Oxidation ditch
W_HZ_LA_2	Hangzhou	HZ	Lin’an	LA	Zhejiang	Oxidation ditch
W_HZ_QG_1	Hangzhou	HZ	Qige	QG	Zhejiang	Anaerobic-Anoxic-Oxic (A2/O)
W_HZ_QG_2	Hangzhou	HZ	Qige	QG	Zhejiang	Anaerobic-Anoxic-Oxic (A2/O)
W_LS_1#_1	Lasa	LS	1#	1#	Tibet	Anaerobic-Anoxic-Oxic (A2/O)
W_LS_1#_2	Lasa	LS	1#	1#	Tibet	Anaerobic-Anoxic-Oxic (A2/O)
W_LS_2#_1	Lasa	LS	2#	2#	Tibet	Anaerobic-Anoxic-Oxic (A2/O)
W_LS_2#_2	Lasa	LS	2#	2#	Tibet	Anaerobic-Anoxic-Oxic (A2/O)
W_LZ_AN_1	Lanzhou	LZ	An’ning	AN	Gansu	Sequence Batch Reactor (SRB)
W_LZ_AN_2	Lanzhou	LZ	An’ning	AN	Gansu	Sequence Batch Reactor (SRB)
W_NJ_DC_1	Nanjing	NJ	Dachang	DC	Jiangsu	Anaerobic-Anoxic-Oxic (A2/O)
W_NJ_DC_2	Nanjing	NJ	Dachang	DC	Jiangsu	Anaerobic-Anoxic-Oxic (A2/O)
W_NJ_JXZ_1	Nanjing	NJ	Jiangxinzhou	JXZ	Jiangsu	Anaerobic-Anoxic-Oxic (A2/O)
W_NJ_JXZ_2	Nanjing	NJ	Jiangxinzhou	JXZ	Jiangsu	Anaerobic-Anoxic-Oxic (A2/O)
W_NN_JN_1	Nanning	NN	Jiangnan	JN	Guangxi	Anaerobic-Anoxic-Oxic (A2/O)
W_NN_JN_2	Nanning	NN	Jiangnan	JN	Guangxi	Anaerobic-Anoxic-Oxic (A2/O)
W_NN_LD_1	Nanning	NN	Langdong	LD	Guangxi	Sequence Batch Reactor (SRB)
W_NN_LD_2	Nanning	NN	Langdong	LD	Guangxi	Sequence Batch Reactor (SRB)
W_SH_MH_1	Shanghai	SH	Minghang	MH	Shanghai	Anaerobic-Anoxic-Oxic (A2/O)
W_SH_MH_2	Shanghai	SH	Minghang	MH	Shanghai	Anaerobic-Anoxic-Oxic (A2/O)
W_SH_MHSZ_1	Shanghai	SH	Minghangshuizhi	MHSZ	Shanghai	Anaerobic-Anoxic-Oxic (A2/O)
W_SH_MHSZ_2	Shanghai	SH	Minghangshuizhi	MHSZ	Shanghai	Anaerobic-Anoxic-Oxic (A2/O)
W_SZ_GM_1	Shenzhen	SZ	Guangming	GM	Guangdong	Anaerobic-Anoxic-Oxic (A2/O)
W_SZ_GM_2	Shenzhen	SZ	Guangming	GM	Guangdong	Anaerobic-Anoxic-Oxic (A2/O)
W_SZ_LF_1	Shenzhen	SZ	Luofang	LF	Guangdong	Oxidation ditch
W_SZ_LF_2	Shenzhen	SZ	Luofang	LF	Guangdong	Oxidation ditch
W_TJ_XYL_1	Tianjin	TJ	Xianyanglu	XYL	Tianjin	Anoxic/Oxic (A/O)
W_TJ_XYL_2	Tianjin	TJ	Xianyanglu	XYL	Tianjin	Anoxic/Oxic (A/O)
W_TJ_ZGZ_1	Tianjin	TJ	Zhangguizhuang	ZGZ	Tianjin	Anoxic/Oxic (A/O)
W_TJ_ZGZ_2	Tianjin	TJ	Zhangguizhuang	ZGZ	Tianjin	Anoxic/Oxic (A/O)
W_WH_NTZ_1	Wuhan	WH	Nantaizi	NTZ	Hubei	Carrousel oxidation ditch
W_WH_NTZ_2	Wuhan	WH	Nantaizi	NTZ	Hubei	Carrousel oxidation ditch
W_WH_TXH_1	Wuhan	WH	Tangxunhu	TXH	Hubei	DE oxidation ditch
W_WH_TXH_2	Wuhan	WH	Tangxunhu	TXH	Hubei	DE oxidation ditch
W_WH_TXH_22	Wuhan	WH	Tangxunhu	TXH	Hubei	DE oxidation ditch
W_WLMQ_HD_1	Wulumuqi	WLMQ	Hedong	HD	Sinkiang	Sequence Batch Reactor (SRB)
W_WLMQ_HD_2	Wulumuqi	WLMQ	Hedong	HD	Sinkiang	Sequence Batch Reactor (SRB)
W_WLMQ_HX_1	Wulumuqi	WLMQ	Hexi	HX	Sinkiang	Sequence Batch Reactor (SRB)
W_WLMQ_HX_2	Wulumuqi	WLMQ	Hexi	HX	Sinkiang	Sequence Batch Reactor (SRB)
W_XA_SW_1	Xi’an	XA	Sanwu	SW	Shan’xi	Orbal oxidation ditch
W_XA_SW_2	Xi’an	XA	Sanwu	SW	Shan’xi	Orbal oxidation ditch
W_XA_WW_1	Xi’an	XA	Wuwu	WW	Shan’xi	Anaerobic-Anoxic-Oxic (A2/O)
W_XA_WW_2	Xi’an	XA	Wuwu	WW	Shan’xi	Anaerobic-Anoxic-Oxic (A2/O)

^a^Format of sample name: X(season)_XX(abrr. of city)_XXX(code for WWTP)_X(day), where S/W in season represents for summer/winter and 1/2 in day stands for day 1/day 2

Genomic DNA was extracted from the collected pellets using the FastDNA® Spin kit for Soil (MP Biomedicals, France) following the manufacturer’s instructions. Total DNA was eluted in 100 μL of sterile water and kept at −20 °C until use. DNA concentrations and purity were measured using a NanoDrop spectrophotometer (ND-1000, Nanodrop, USA) [16].

### DNA sequencing

The hypervariable V4-V5 region of the 16S rRNA gene was amplified using the primer pair (515 F: 5′-GTGCCAGCMGCCGCGG-3′ and 907R: 5′-CCGTCAATTCMTTTRAGTTT-3′ with sample-identifying six-nucleotide barcodes) [17]. The 4 × 50 μL reaction system was set up for each PCR amplification under the following program: initial denaturation at 95 °C for 5 min, and 30 cycles at 95 °C for 30 s, 58 °C for 30 s, and 72 °C for 30 s and a final extension at 72 °C for 10 min. The resulting amplicons were purified, quantified, pooled, and sequenced on an Illumina MiSeq PE300 platform (Novogene, Beijing, China). For metagenome sequencing, approximately 3 μg of sewage DNA was used for shotgun library construction with an insert size of 300 bp, followed by Illumina paired-end sequencing on the HiSeq 2500 platform (Novogene, Beijing, China).

### Phylotype analysis

All the raw reads were processed using QIIME [18] (1) to sort and assign by exactly matching the unique barcode into each sample, (2) to remove primers and the sequences with ambiguous bases, primer mismatches, and homopolymers in excess of six bases, or error in barcodes, and (3) to filter low-quality reads with >20 low-quality bases. Chimeric and noisy sequences were also filtered out. After processing, sequences were clustered into operational taxonomic units (OTUs) using Uclust clustering, which groups sequences at a minimum pair-wise identity of 97%. Mitochondrion or chloroplast sequences and singleton OTUs were discarded from the final OTU table. For each resulting OTU, the most abundant read was selected as a representative sequence. The taxonomic classification of each representative sequence was conducted using a Ribosomal Database Project (RDP) Classifier at an 80% confidence threshold (Version 2.2) [19, 20]. Alignment of the OTU representative sequences was conducted using a PyNAST aligner [21], and a phylogenetic tree was built using a FastTree algorithm [22] for downstream analysis.

Rarefaction was performed to discern Phylogenetic Diversity, Chao1 diversity, Shannon index, and observed species metrics at each sampling depth. To remove the bias caused by different sequencing depth, the OTU table was rarefied and an even sampling depth was set by randomly subsampling the same number of sequences from each sample. Beta-diversity was estimated by computing weighed/unweighed UniFrac and Bray-Curtis distances between every pair of community samples using QIIME.

### Metagenomic analysis

Of the original 116 sewage samples, 24 samples were excluded from metagenomic sequencing due to the low quantities of DNA or poor sequence data (Additional file 1: Table S1). Thus, only 92 samples were further used for metagenomics analysis. Metagenomic sequencing of sewage DNA samples generated 203 Gb pairs of high-quality data with an average of 2.2 Gb for each sample. Data filtration was conducted to remove raw reads with low-quality following the methods used in a previous study [23]. Subsequently, metagenomic sequences were analyzed by BLASTx against the Structured Non-redundant Clean Antibiotic Resistance Genes Database (SNC-ARDB) with *E* value ≤1 × 10^−5^. A read was annotated to be a resistance gene if its BLAST hit for the alignment against SNC-ARDB had ≥90% amino acid read identity for ≥25 amino acids [16, 24]. In the present study, a package of customized scripts was developed to automatically classify the BLAST hits into different types and subtypes of resistance genes. The detailed procedure for sorting sequences using a customized Python script was reported previously [23].

SNC-ARDB contains a number of genes for efflux proteins that do not necessarily confer resistance phenotypes. These proteins do, however, function in the efflux of antibiotics and have previously been classified as resistance genes and in the Comprehensive Antibiotic Resistance Database (CARD) [25–27]. Therefore, efflux pump-related genes were retained in the SNC-ARDB to evaluate antibiotic resistance potential [16].

The ‘abundance’ of the resistance type or subtype was calculated as previously reported by Li et al. (2015) [16]. Thus, the abundance of resistance genes based on metagenomic analysis was compared with those derived from qPCR in the previous studies. The abundance of resistance genes was transformed to ‘concentration’ (copies per liter) by normalization to the absolute copy number of 16S rRNA gene [28]. The average copy number of 16S rRNA genes per bacterium is currently estimated at 4.1 based on the Ribosomal RNA Operon Copy Number Database (rrnDB version 4.4.4) [29]. The numbers of bacterial cells were calculated by dividing the copy number of 16S rRNA gene by 4.1, and the ‘relative abundance’ of resistance genes (copies per bacterial cell) was estimated by dividing the ARGs concentration in each sample by its corresponding number of bacterial cells. Additionally, the copy number of resistance genes discharged by each person per day in urban areas is defined as ‘ARG load’, which can be calculated by the formula: ARG load (copies/capita/day) = (the average concentration of sewage ARGs) × (the volume of municipal sewage discharge)/urban population. ‘ARG burden’ (copies/day, the total ARG load in urban areas) is calculated by multiplying the medium value of ARG load by total urban population.

### Real-time qPCR quantification of 16S rRNA gene

Real-time qPCR assay of total bacteria was performed using a SYBR® Green approach on a Roche 480 (Roche Inc., USA). The absolute copy numbers of 16S rRNA gene were quantified using primers 515 F and 907R. The qPCR system (20 μL) amplification was conducted as reported previously [30]. The size of amplified fragments was about 410 bp. For the preparation of 16S rRNA gene standards, 16S rRNA gene was amplified from extracted DNA and then was cloned into the pMD 19-T vector (TaKaRa, Japan). Plasmids containing the target gene were used as standards for the calibration curve. All qPCR assays were conducted in triplicate with negative and positive controls.

### Human gut microbiome analysis

In this study, human gut microbiota was defined as the bacterial genera detected in human gut or human intestinal tract. To track the human gut microbiome fingerprint in the sewage, the human gut microbiome database including 382 bacterial genera (Additional file 2: Dataset 1) was retrieved from Human Microbiome Project (HMP) [31] and the Metagenomics of the Human Intestinal Tract (MetaHit) [32] project. The microbial catalogue reference set (16S rRNA gene) from these two human metagenomic projects covers almost all genera of human gut bacteria and is a useful resource for further analyses of human gut microbiome [33].

### Statistical analysis and network analysis

Averages and standard deviations were determined using Excel 2010 (Microsoft Office 2010, Microsoft, USA). One-way analysis of variation (ANOVA), paired-sample *t* tests and correlation tests were performed using SPSS V20.0 (IBM, USA). All statistical tests were considered significant at *P* < 0.05. Diversity index, non-metric multidimensional scaling (NMDS) and significance test (Adonis test, procrustes analysis, and mantel test) were performed in R 3.1.0 with vegan 2.2.0 [34, 35]. Post-hoc plot was generated using STAMP V2.1.3 [36]. To investigate co-occurrence patterns of microbial community and resistome, correlation matrixes were constructed by calculating each pairwise Spearman’s rank correlations. The *P* value was adjusted with a multiple testing correction using FDR method to reduce the false-positive results [37]. A correlation between any two items was considered statistically robust if the Spearman’s correlation coefficient (*ρ*) was > 0.7 and the *P* value was < 0.01 [16, 38]. The resulting correlation matrixes were translated into an association network using Gephi 0.9.1 [39]. An informatics mathematical approach based on geographical information systems, ArcGIS, was applied to map the resistance load and the resistance burden at varying spatial scales [40]. Spatial autocorrelation analysis was conducted to evaluate spatial dependency of ARG burdens between provinces using ArcGIS [41].

## Results

### Diversity and abundance of the resistome in urban sewage

**Fig. 1:**
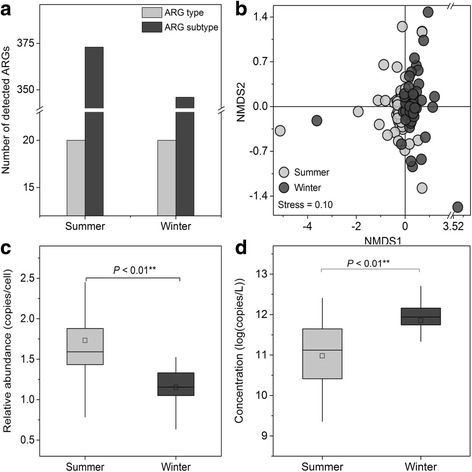
The overall profile of antibiotic resistance genes from urban sewages in China. **a** The number of detected ARG types and subtypes in sewages. **b** Non-metric multidimensional scaling (NMDS) analysis based on the abundance of ARGs (copy of ARG per copy of 16S rRNA gene) showing the seasonal variation of ARGs (Adonis test, *P* < 0.01). NMDS analysis was conducted using Bray-Curtis distance. **c** Relative abundance of ARGs presented as copy number of ARGs per bacterial cell. **d** The concentration of ARGs in sewages presented using the sum of copy numbers of detected mobile genetic elements (MGEs) and ARGs conferring resistance to a specific class of antibiotics. Mean ± SD; ANOVA; *P < 0.05; **P < 0.01

Twenty resistance gene types consisting of 381 subtypes were detected, with 373 subtypes in summer samples and 346 subtypes in winter samples, respectively (Fig. 1a). The three most dominant resistance gene types, conferring aminoglycoside, tetracycline, and beta-lactam resistance, accounted for 54.1% of the total ARG abundance (Additional file 3: Figure S1a). For the resistance gene subtypes, genes encoding beta-lactamase, sulfonamide (*sul*I), and tetracycline (*tet*40) were most common across all sewage (Additional file 4: Dataset 2). Resistance gene profiles indicated distinct seasonal clustering (Adonis test, *P* < 0.01) (Fig. 1b) and paired-samples *t* tests further demonstrated significant seasonal differences within most cities (*P* < 0.05), except the cities of Shenzhen (SZ), Tianjin (TJ), and Xi’an (XA) (Additional file 5: Table S2).

The relative abundance of antibitotic resistance genes in summer (1.73 copies per bacterial cell) was significantly higher than that in winter (1.15 copies per bacterial cell) (*P* < 0.01) (Fig. 1c and Additional file 3: Figure S1b). In terms of abundance, winter samples contained highly abundant bacteria (1.21 × 10^12^ cells/L) (*P* < 0.01) and resistance genes (1.79 × 10^12^ copies/L) (*P* < 0.01), while summer samples were found to harbor lower bacterial abundance (1.70 × 10^11^ cells/L) and lower resistance gene concentration (3.27 × 10^11^ copies/L) (Fig. 1d, Additional file 3: Figure S1c and Figure S1d). Significantly different seasonal abundances were observed for 27 ARG subtypes (Additional file 6: Figure s2a).

### Geographical burden of ARGs in Chinese urban sewage

**Fig. 2:**
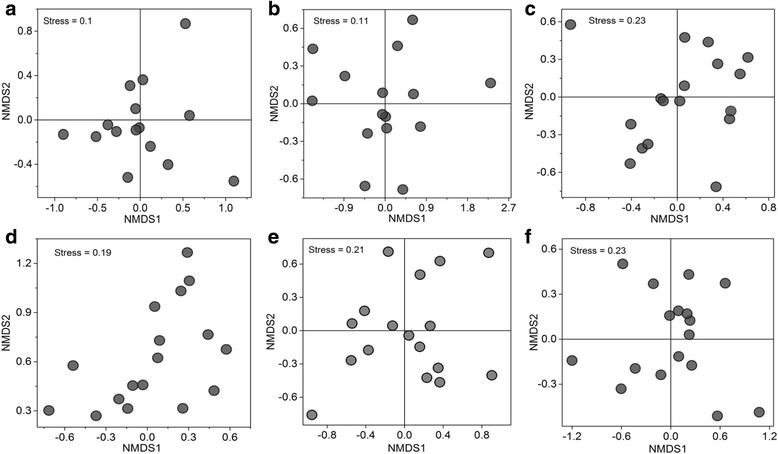
Non-metric multidimensional scaling (NMDS) analysis depicting geographical distribution of antibiotic resistomes using Bray-Curtis distance (**a**, total ARGs; **b**, shared ARGs) and microbial communities (**c**, total OTUs; **d**, shared OTUs; **e**, human gut microbiota; **f**, shared human gut microbiota) of urban sewages (mean ± SD)

**Fig. 3:**
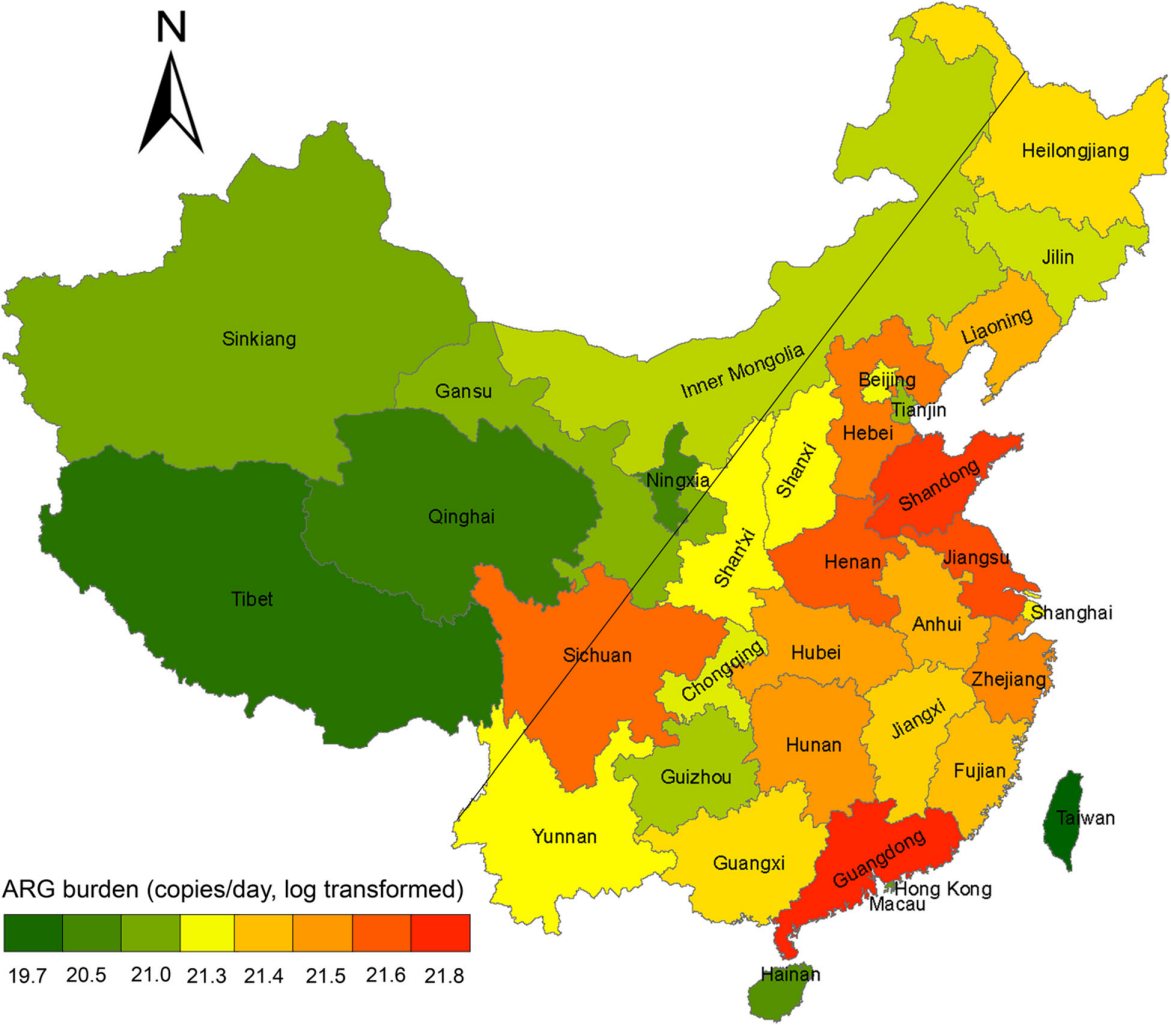
ArcGIS map showing the ARG burden based on urban populations of administrative districts in China. The black line on the map refers to the Chinese demographic “Hu Huanyong line”. The value presented in the legend was log transformed ARG burden (copies/day) discharged by urban populations. The ARG burden in Chinese administrative regions is calculated by multiplying the medium value of ARG load (9.47 × 10^13^ copies/cap/day) by total urban population

No distinct regional distribution pattern of the antibiotic resistome was observed among the sewage samples from different cities (Additional file 7: Figures S3a and S3b and Fig. 2a, b). Based on the demographic data (Additional file 8: Dataset 3), the total volume of domestic sewage discharge ranged from 0.329 to 7.846 million tons/day across major Chinese cities in 2014. The ARG load in the major Chinese cities was calculated with a range from 5.89 × 10^12^ to 7.85 × 10^14^ copies/person/day (Additional file 9: Figure S4). The urban ARG burden in Chinese administrative regions was calculated by multiplying the median value (9.47 × 10^13^ copies/person/day) of ARG load by the urban population, resulting in a range from 5.40 × 10^19^ to 6.91 × 10^21^ copies (Additional file 8: Dataset 3). ArcGIS mapping of antibiotic resistance showed significantly higher ARG burden in the east of China, which was 1–2 orders of magnitude higher than those in the west of China. The antibiotic resistance distribution was distinguished by the “Hu Huanyong line”, which delineates a striking difference in the distribution of China’s population (Fig. 3). A strong spatial dependency was observed in the ARG burdens between geographically nearby provinces with Moran’s I index of 0.173 (variance = 0.0056; *z* score = 2.709; *p* value = 0.007). Moran’s I index > 0 indicates spatial autocorrelation and larger values of Moran’s I indicate higher spatial autocorrelation. *P* value < 0.01 indicates an extremely significant spatial autocorrelation.

### Characterization of bacterial communities in urban sewage

**Fig. 4:**
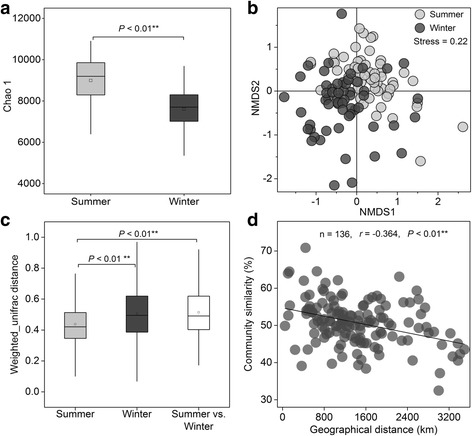
Overall profile of microbial diversity of the sewage samples from Chinese WWTPs. Diversity of the microbiome was evaluated by using OTUs with 97% similarity cutoffs. **a** Rarefied Chao 1 index at a sequencing depth of 22,149 showing significant difference of α-diversity between summer and winter samples (mean ± SD; ANOVA; *P < 0.05; **P < 0.01). **b** NMDS analysis showing the overall pattern of microbial communities (Adonis test, *P* < 0.01). **c** β-diversity of microbial communities computed with weighted UniFrac indices within/between the summer and winter samples (mean ± SD; ANOVA; *P < 0.05; **P < 0.01.). **d** Spearman’s rank correlations between the Bray-Curtis similarity of microbial communities and geographical distance (*n* is the number of comparison)

From PCR amplicons spanning the V4 and V5 hypervariable regions of the 16S rRNA gene, 6,174,489 high quality sequences (22,149–147,635 per sample) were clustered into 74,138 OTUs (2551–10,691 for each sample, mean = 5,509) (Additional file 1: Table S1). Higher OTU numbers and higher microbial diversity (Chao 1 index, *P* < 0.01) (Fig. 4a) were observed in summer sewage. Overall, microbial cohorts closely clustered by sampling time (Adonis test, *P* < 0.01) (Fig. 4b) and sewage microbiomes between seasons were more heterogeneous than those within either season (*t* test, *P* < 0.01) (Fig. 4c). A significant distance-decay effect was also observed—similarity in microbial communities between any two cities decreased with increasing geographic distance (*r* = −0.364, *P* < 0.01) (Fig. 4d). Similar to antibiotic resistome, no geographical cluster of either bacterial community or shared bacterial OTUs was also observed (Additional file 7: Figures S3c and S3d and Fig. 2c, d).

*Proteobacteria*, *Bacteroidetes*, *Firmicutes*, and *Fusobacteria* were the dominant phyla, accounting for 58.7 to 98.5% of sequences within the 116 samples (Additional file 10: Figure S5a). *Bacteroidetes* had significantly higher abundance in summer samples than that in winter samples (*P* < 0.01), while both *Firmicutes* and *Actinobacteria* were more abundant in winter than those in summer (Additional file 10: Figure S5b). Significantly different seasonal abundances were also observed in several bacterial classes, for example, *Deltaproteobacteria*, *Bacteroidia*, and *Clostridia* (Additional file 6: Figure S2b). At the genus level, the most abundant 30 genera were mainly classified to *Proteobacteria*, *Bacteroidetes*, and *Firmicutes*, and *Bacteroides*, *Prevotella* and *Acinetobacter* were the top 3 abundant genera (Additional file 11: Figure S6a).

### Core resistome and microbiome in Chinese urban sewage

**Fig. 5:**
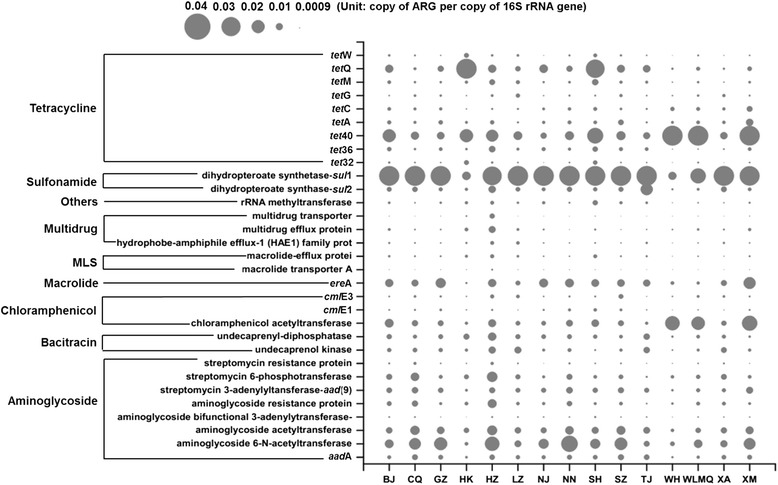
Bubble graph showing the abundance (copy of ARG per copy of 16S rRNA gene) of the core resistome, which was shared by all sewage samples. *MLS* Macrolide-Lincosamide-Streptogramin resistance. Others, the genes coding other unclassified antibiotic resistance proteins or other functional proteins

128 resistance genes were shared in more than 80% of samples, accounting for 95.6% of all the ARGs observed. A set of 31 resistance genes were found in all sewage samples, and this core resistome contributed 57.7% (ranging from 29.4 to 84.3%) to the total ARGs detected (Fig. 5). Among the core resistome, the genes for aminoglycoside, tetracycline, beta-lactam, and MLS resistance were dominant (Additional file 12: Figure S7a). Resistance genes *sul*I, *tet*40, and one encoding chloramphenicol acetyltransferase were the most abundant resistance subtypes (Fig. 5). The core resistome clustered by season (Adonis test, *P* < 0.01) (Additional file 12: Figure S7b), but geographical clustering was not observed (Additional file 7: Figure S3 and Fig. 2b).

**Fig. 6:**
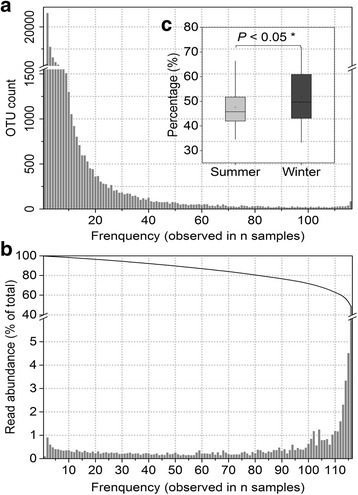
Frequency distribution of OTUs across samples. **a** The number of OTUs commonly observed at each frequency (in n samples). **b** The *bar* presents the read abundance of OTUs observed at each frequency (in n samples) and the *line* denotes the cumulative total these frequencies from the most to least frequently observed. **c** The embedded plot revealing the percentage of human gut bacteria in the shared OTUs based on the number of sequences

Common 16S rRNA OTUs accounted for 64.6% of the total reads in more than 100 samples. A highly shared prevalence of microbiome among all sewage samples was also observed to include 88 classified OTUs, and accounted for 13.6–67.7% (on average 48.8%) of the total bacterial abundance in each sample (Fig. 6a, b). The core OTUs belonged to 33 dominant genera that were affiliated to 7 phyla (Additional file 12: Figure S7c), of which *Proteobacteria*, *Bacteroidetes*, and *Firmicutes* were the most abundant. At the genus level, the most prevalent OTUs were *Bacteroidetes*, *Prevotella*, and *Trichococcus* (Additional file 11: Figure S6b). Similar to the core resistome profiles, these shared OTUs were separated by season without a geographical distribution pattern (Fig. 2d and Additional file 12: Figure S7d).

### Linking antibiotic resistome with bacterial phylogeny in Chinese urban sewage

**Fig. 7:**
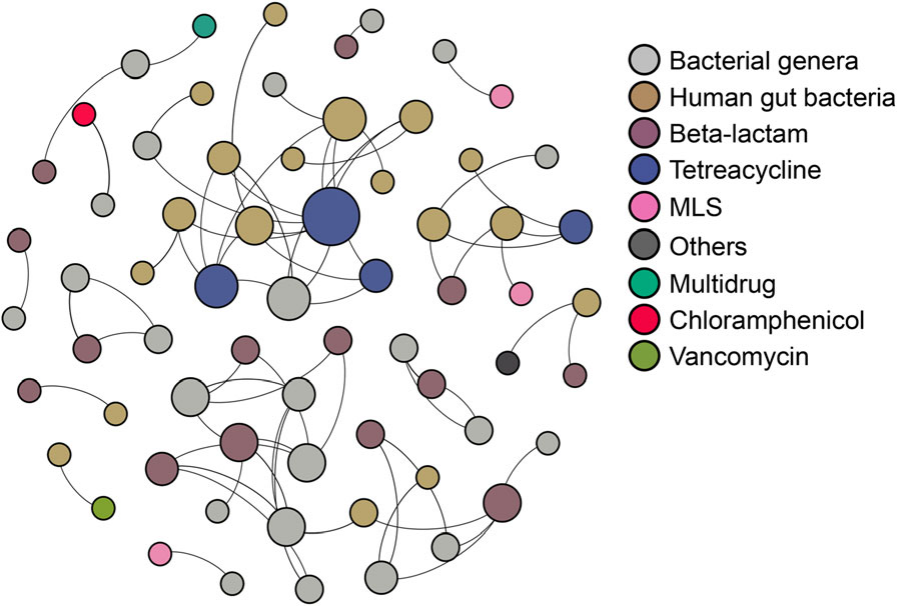
Co-occurrence network analysis showing the correlation between resistance genes and bacterial taxa at genus level. Only connections with a strong (Spearman’s *ρ* > 0.7) and significant (*P* value < 0.01) correlation were presented in the network. The size of the nodes (*circles*) is proportioned to the number of connections (the *degree*), and the width of the edges (*lines* connecting the circles) is proportioned to the Spearman’s correlation coefficient between bacterial genera and ARGs. *MLS* Macrolide-Lincosamide-Streptogramin resistance. Others, the genes coding other unclassified antibiotic resistance proteins or other functional proteins

Co-occurrence patterns between antibiotic resistance and bacterial assemblages were explored based on strong (*ρ* > 0.7) and significant (*P* < 0.01) correlations (Fig. 7). There were more complex and dense correlations between bacterial communities in winter than those in summer, whereas looser relationships with the antibiotic resistome were observed in summer (Additional file 13: Figures S8). Co-occurrence, using network analysis, is summarized in Additional file 14: Dataset 4. Mantel test indicated that the antibiotic resistome was significantly correlated with bacterial phylogeny (*P* < 0.01). Exploration of connections between ARGs and bacterial genera showed that most genera from the same phylum had similar antibiotic resistance profiles (Additional file 15: Table S3), but this does not prove that bacterial OTUs were actually hosts of resistance genes. In addition, significant correlation between core resistance genes and core bacterial OTUs was also observed (Procrustes test, *M* ^2^ = 0.927, *P* < 0.001, 9999 permutations).

### Human gut microbiota in urban sewage

A total of 205 genera of gut bacteria were detected in 116 samples with abundance ranging from 6.1 to 59.9% (average 28.6%) in each sample. Both the diversity and the relative abundance of gut bacteria in winter samples were higher than those in summer samples (Additional file 11: Figure S6e). A significant seasonal variation of gut bacterial community structure was also observed (Adonis test, *P* < 0.01) (Additional file 11: Figure S6f). Geographical profiles varied from city to city, with no obvious regional clustering in either total gut bacteria or shared gut bacteria (Fig. 2e, f). Of the detected human gut microbiota, *Proteobacteria*, *Firmicutes*, and *Bacteroidetes* were most abundant phyla. The most abundant gut bacterial genera in sewage were affiliated with *Acinetobacter*, *Arcobacter*, and *Paludibacter* (Additional file 11: Figure S6c). The shared human gut microbiota, including 32 human gut genera detected in all sewage accounted for 49.6% (ranging from 34.6 to 70%) of the shared OTUs (Fig. 6c).

## Discussion

The urban sewage resistome represents the emission of antibiotic resistance from the gastrointestinal tracts of citizens into wastewater treatment plants. A large-scale sampling of municipal sewage from 17 major cities across China was performed in this study. The seasonal variation and geographical distribution of the urban sewage antibiotic resistome were characterized. Municipal sewage harbored diverse and abundant resistance genes, conferring resistance to almost all antibiotics, highlighting that municipal sewage could be a major conduit for transferring antibiotic resistance genes into the environment. Significant seasonal differences were observed in the urban sewage antibiotic resistome (*P* < 0.01). Seasonal temperature changes might have a significant influence on the variation of antibiotic resistance and on the composition of microbial community. The seasonality of microbial communities in lakes [42], soil [43], and sludge [44] has been well documented. Temperature and temperature-dependent organic matter load can foster the proliferation of microbial taxa that carry resistance genes, or improve the growth of non-resistant microorganisms [45]. Antibiotic administration could also explain the clear seasonality in shifts within the sewage resistome [3, 12]. Seasonal variation in antibiotic consumption driven by associated seasonality in pathologies exerts selective pressure, leading to selection and subsequent dissemination of antibiotic resistance genes in wastewater [3]. Thus, seasonally variable release of antibiotics, bacteria, and resistance genes into municipal sewage can alter bacterial populations and remodel their resistome [46, 47]. A recent study on sewers further supported the speculated reason, demonstrating a clear seasonal pattern in the relative abundances of resistance genes, and that this coincided with the overall rates of antibiotic prescription [12].

Higher concentrations of ARGs were detected in winter sewage, being approximately one order of magnitude higher than those of summer. This finding was supported by a recent study, where increases in ARGs in sewers were always encountered in colder seasons of the year, when the more frequent seasonal epidemic diseases contributed to the therapeutic prescription of antibiotics [12]. In addition, dilution of urban sewages by increasing domestic daily water discharges in summer may be another explanation for lower ARG concentration in summer. Similar observations were found in urban streams, where total bacterial numbers were the highest in winter [48, 49]. Although ARGs absolute concentrations in winter sewage were greater, a significantly higher relative abundance of ARGs in summer sewage was observed. The major reason for such disparity might be that the bacterial density in winter sewage (1.21 × 10^12^ cells/L) was much greater than that in summer sewage (1.70 × 10^11^ cells/L). Bacterial biomass in sewage has been quantified using flow cytometry within the range from 10^10^ to 10^12^ cells/L [29, 50], and this is consistent with our results when normalizing for 16S rRNA gene copy number per cell.

The total output of resistance genes was estimated by considering the amount of domestic sewage and urban populations in Chinese administrative districts to quantify the regional ARG burden at a national scale. A strong spatial dependency in the distribution of ARG abundance in various administrative areas were observed, with two main regions separated by the demographic “Hu Huanyong line”, which is based on climatic zonation and population density [51]. It was previously reported that industrialization was correlated with the antibiotic resistance burden of the human gut, and this was in turn driven by age, diet, cultural tradition, climate, pathogen carriage, and periodic perturbation, for example, by antibiotic exposure [10, 52]. A similar geographic distribution was found in the antibiotic emission densities in Chinese river basins [40], suggesting that human activities are the major driver of resistance gene distribution.

Geographical clustering was not observed in the structure of either antibiotic resistome or bacterial community in Chinese sewage. The core antibiotic resistome and the core microbial community were stable across WWTPs [23, 53]. The resistome closely correlates with host-related bacterial phylogeny in sewage [12], indicating that the shared resistome and the core microbiota might play a vital role in the profile of urban resistome and its microbial community. Despite no distinct geographical grouping, there was a distance-decay effect in the similarity in bacterial community composition. This has been specifically reported in freshwater bacterial communities, phyllosphere bacteria, and more generally [54, 55].

Median fecal dry mass production is estimated at 29 g per person per day [56, 57]; and human intestinal contents range from 10^10^ to 10^11^ bacterial cells per gram (dry weight) [58]. Therefore, it was estimated that approximately 10^11^ ~ 10^12^ bacterial cells per person per day were discharged into sewage. Given the proportion of these cells that carry antibiotic resistance, there are clear pathways for dissemination of resistance genes via sewage [59]. Although the core resistome was shared by all populations investigated here, there were differences in the abundance of ARGs between urban areas. This suggests that monitoring sewage systems for ARGs could provide a real-time estimate of antibiotic resistance threats in specific areas, and this in turn could be used to inform treatments and to promote stewardship of antibiotics.

## Conclusions

Currently, sound and necessary data on seasonal and geographical characterization of antibiotic resistome in urban sewage is still lacking. This study provided solid evidence for seasonal and geographical patterns of the profiles of antibiotic resistome and potential ARG hosts via a national-scale survey. Seasonal variation in both antibiotic resistomes and bacterial communities was observed in urban sewage. No distinct geographical cluster was found in the distribution of the resistance genes and bacterial community composition. The demographic “Hu Huanyong line” separated the regional ARG burden into two main regions, suggesting human activities might be the major driver of antibiotic resistance burden distribution. A core, shared antibiotic resistome accounted for more than 50% of the total resistance genes, and was significantly associated with the core microbial community. The shared resistome and the shared bacterial community exhibited a distinct seasonal distribution, but did not show geographical clusters, indicating that the share resistome and the core microbiota might play a vital role in the profile of urban resistome and its microbial community. In addition, the strong correlations between resistome and bacterial communities, especially between the core, shared resistome and the core human gut microbiota, indicated the contribution of human gut microbiota to the dissemination of antibiotic resistance. These data provide dynamic background (seasonal and geographical variation) for mitigation activities in WWTPs based on the presence of ARGs and practical guide for improving antibiotic management in the urban sewage.

## Additional files

Additional file 1: **Table S1.** Supplementary information of WWTP sewage samples. (XLSX 16 kb)

Additional file 2: **Dataset 1.** List of human gut bacterial genera. (XLSX 15 kb)

Additional file 3: **Figure S1.** Composition of ARG types in summer and winter sewages based on the abundance (**a**, copy of ARG per copy of 16S rRNA gene), the relative abundance (**b**, copies/cell), and the concentration (**c**, copies/L) of ARGs. The embedded chart (**d**) revealed the significant difference (*P* < 0.01**) in bacterial copy numbers between summer and winter. The difference in the liter-based cells between summer and winter was significant statistically. *MLS* Macrolide-Lincosamide-Streptogramin resistance. Others, the ARG types with the average abundance less than 0.01 copies of ARG per copy of 16S rRNA gene. ANOVA, * represents *P* < 0.05, and ** represents *P* < 0.01. (PDF 165 kb)

Additional file 4: **Dataset 2.** The abundance of top 10 ARG subtypes in urban sewages. (XLSX 24 kb)

Additional file 5: **Table S2.** Paired samples *t* test (Student’s t test) showing the significant seasonal differences of ARG profile from the same city. *P* < 0.05 represents a significant difference. (XLSX 10 kb)

Additional file 6: **Figure S2.** Post-hoc plot (ANOVA, null hypothesis) indicating the mean proportion of the dominant ARG subtypes (**a**) and bacterial classes (**b**) with significant differences (adjusted *P* < 0.05, FDR adjusted) between summer and winter. (PDF 329 kb)

Additional file 7: **Figure S3.** Cluster analysis (hierarchical cluster) based on between-groups linkage method revealed that no distinct geographic clustering of resistome and bacterial community in urban sewage was observed. The Euclidian distances between two observations were measured using interval and square Euclidean distance. **a** All ARG subtypes. **b** Shared ARG subtypes. **c** Bacterial community. **d** Shared bacterial OTUs. **e** Human gut microbiota. **f** Shared human gut genera. *Numbers* on the top indicate rescaled distance cluster combine. (PDF 220 kb)

Additional file 8: **Dataset 3.** Urban ARG load of 15 major Chinese cities and urban ARG burden of Chinese administrative regions in 2014. (XLSX 12 kb)

Additional file 9: **Figure S4.** ARG load and amount of domestic sewage discharged in major Chinese cities. The base map used is from the National Fundamental Geographic Information System of China. The *red* and *black* columns represent the average ARG load (copies/capita/day) and the total amount of domestic sewage discharged in each city (tons/day), respectively. All of the values marked in the map were standardized by log algorithm. (PDF 323 kb)

Additional file 10: **Figure S5.** Bacterial community compositions in sewage samples. **a** The relative abundance of major phyla in each city. **b** Comparison of the relative abundance of major phyla between summer and winter samples at the phylum level. ANOVA, * represents *P* < 0.05, and ** represents *P* < 0.01. Others refer to the phyla with the average percentage less than 2%. (PDF 230 kb)

Additional file 11: **Figure S6.** Relative abundances of the bacterial genera in all the WWTP sewage samples. Genera are colored by their respective phylum. **a** The relative abundance of the top 30 abundant genera, representing as the relative abundance of each genus. **b** The relative abundance of the 33 shared, classified bacterial genera. **c** The relative abundance of top 30 human gut microbial genera. (PDF 559 kb)

Additional file 12: **Figure S7.** Seasonal variation of antibiotic resistomes and microbial communities in sewage. **a** Percentage of the core resistomes in urban sewage samples. *MLS* Macrolide-Lincosamide-Streptogramin resistance. **b** NMDS analysis revealing the distribution pattern of the core resistomes at subtype level (Adonis tests, *P* < 0.01). **c** Relative abundance of the phyla that shared OTUs were affiliated to in WWTP sewages. **d** NMDS analysis of shared OTUs with seasonal change as instrumental variables based on the abundance of OTUs across 116 individual sewage samples (Adonis test, *P* < 0.01). **e** Percentage of human gut microbial phyla with the relative abundance in all of the WWTP sewages. **f** NMDS analysis revealing the pattern of human gut bacteria with the seasonal change (Adonis test, *P* < 0.01). ANOVA, * represents *P* < 0.05, and ** represents *P* < 0.01. NMDS analysis was conducted based on Bray-Curtis distance. (PDF 436 kb)

Additional file 13: **Figure S8.** Co-occurrence patterns of summer (**a)** and winter (**b**) antibiotic resistome and summer (**c)** and winter (**d**) microbiome in urban sewages. A connection stands for a strong (Spearman’s *ρ* > 0.7) and significant (*P* value < 0.01) correlation. *Nodes* indicate taxonomic affiliation at ARG subtypes (**a** and **b**) and genus level (**c** and **d**), respectively. The *color* of each node indicates various ARG types (**a** and **b**) and bacterial phyla (**c** and **d**). Size of the nodes was proportioned to the number of connections and the width of the edges (lines connecting the circles) was proportioned to the Spearman’s correlation coefficient. In plot **a** and **b**, *MLS* stands for Macrolide-Lincosamide-Streptogramin resistance and Others represent the genes coding other unclassified antibiotic resistance proteins or other functional proteins; while in plot **c** and **d**, Others refer to the unclassified phyla. (PDF 335 kb)

Additional file 14: **Dataset 4.** Characteristics of network analysis of bacterial taxa and ARGs in summer and winter. (XLSX 35 kb)

Additional file 15: **Table S3.** ARGs host information derived from co-occurrence pattern between the specific ARGs and bacterial taxa. (XLSX 12 kb)

## Abbreviations

ARGs: Antibiotic resistance genes
CARD: Comprehensive Antibiotic Resistance Database
HGT: Horizontal gene transfer
HMP: Human Microbiome Project
MetaHit: Metagenomics of the Human Intestinal Tract
MGEs: Mobile genetic elements
MLS: Macrolide-Lincosamide-Streptogramin resistance
NMDS: Non-metric multidimensional scaling
OTUs: Operational taxonomic units
RDP: Ribosomal Database Project
SNC-ARDB: Structured Non-redundant Clean Antibiotic Resistance Genes Database
WWTPs: Wastewater treatment plants
