# Correction to: Metagenomics of urban sewage identifies an extensively shared antibiotic resistome in China

**DOI:** 10.1186/s40168-018-0504-6

**Published:** 2018-07-09

**Authors:** Jian-Qiang Su, Xin-Li An, Bing Li, Qing-Lin Chen, Michael R. Gillings, Hong Chen, Tong Zhang, Yong-Guan Zhu

**Affiliations:** 1 Key Lab of Urban Environment and Health, Institute of Urban Environment, Chinese Academy of Sciences, 1799 Jimei Road, Xiamen, 361021 China; 2 University of Chinese Academy of Sciences, 19A Yuquan Road, Beijing, 100049 China; 3 State Key Laboratory of Urban and Regional Ecology, Research Center for Eco-Environmental Sciences, Chinese Academy of Sciences, Beijing, 100085 China; 4 Environmental Biotechnology Laboratory, The University of Hong Kong, Pokfulam, Road, Hong Kong, China; 5 Division of Energy & Environment, Graduate School at Shenzhen, Tsinghua University, Shenzhen, 518055 China; 6 Department of Biological Sciences, Macquarie University, Sydney, NSW 2109 Australia; 7 Department of Environmental Engineering, College of Environmental and Resource Sciences, Zhejiang University, Hangzhou, 310058 China

## Correction

Unfortunately, after publication of this article [1], it was noticed that the accession numbers for the study were missing. The correct numbers are PRJNA438554, SRP148953.
