# Supplementary figures and images for: Metagenomics of urban sewage identifies an extensively shared antibiotic resistome in China
Source: Microbiome. 2017 Jul 19;5:84. doi: 10.1186/s40168-017-0298-y (PMC5517792; doi:10.1186/s40168-017-0298-y)

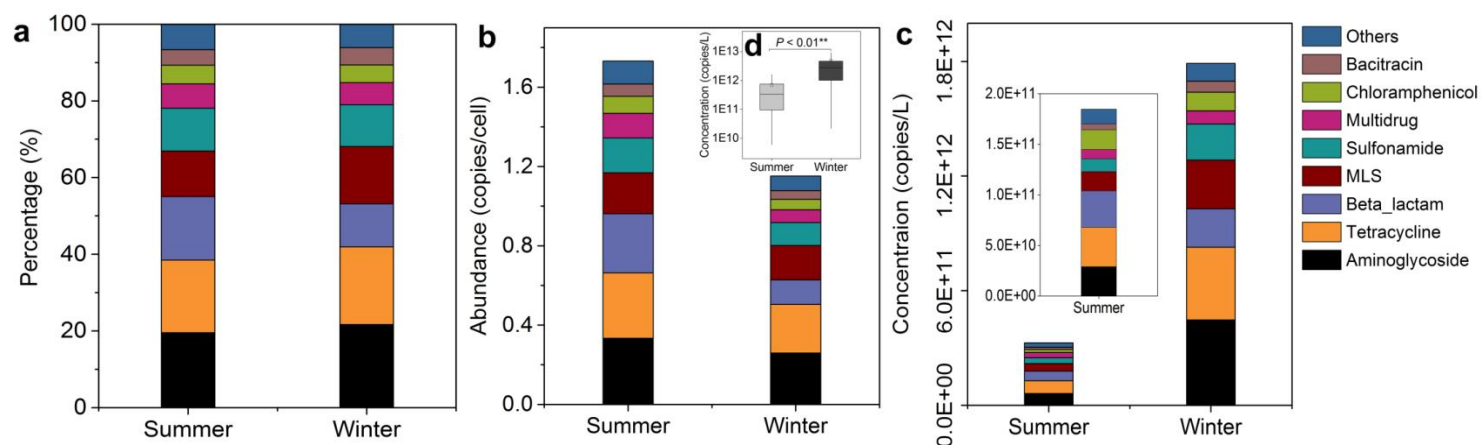

Supplement: Supplementary file 3 — Figure S1. Composition of ARG types in summer and winter sewages based on the abundance (a, copy of ARG per copy of 16S rRNA gene), the relative abundance (b, copies/cell), and the concentration (c, copies/L) of ARGs. The embedded chart (d) revealed the significant difference (P < 0.01**) in bacterial copy numbers between summer and winter. The difference in the liter-based cells between summer and winter was significant statistically. MLS Macrolide-Lincosamide-Streptogramin resistance. Others, the ARG types with the average abundance less than 0.01 copies of ARG per copy of 16S rRNA gene. ANOVA, * represents P < 0.05, and ** represents P < 0.01. (PDF 165 kb) [file 40168_2017_298_MOESM3_ESM.pdf]

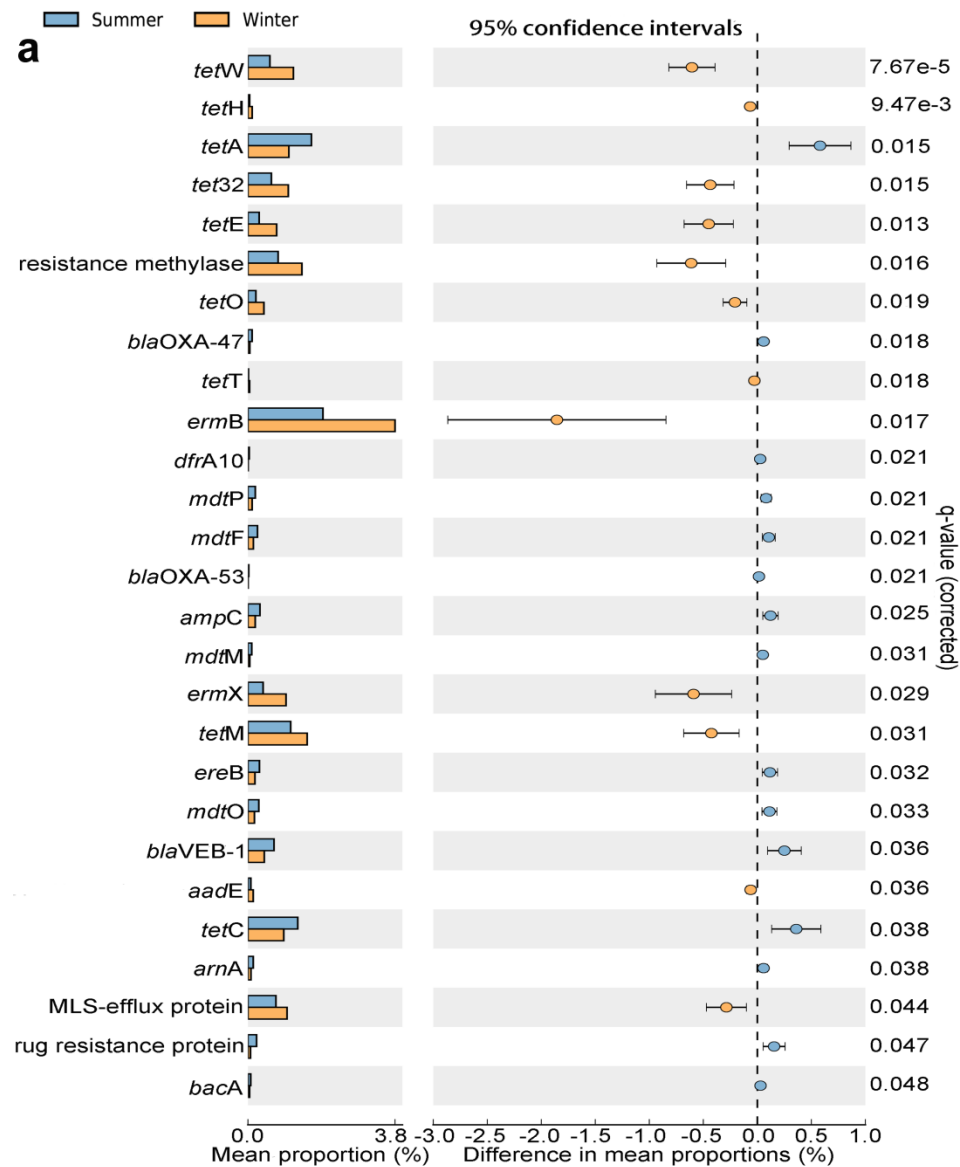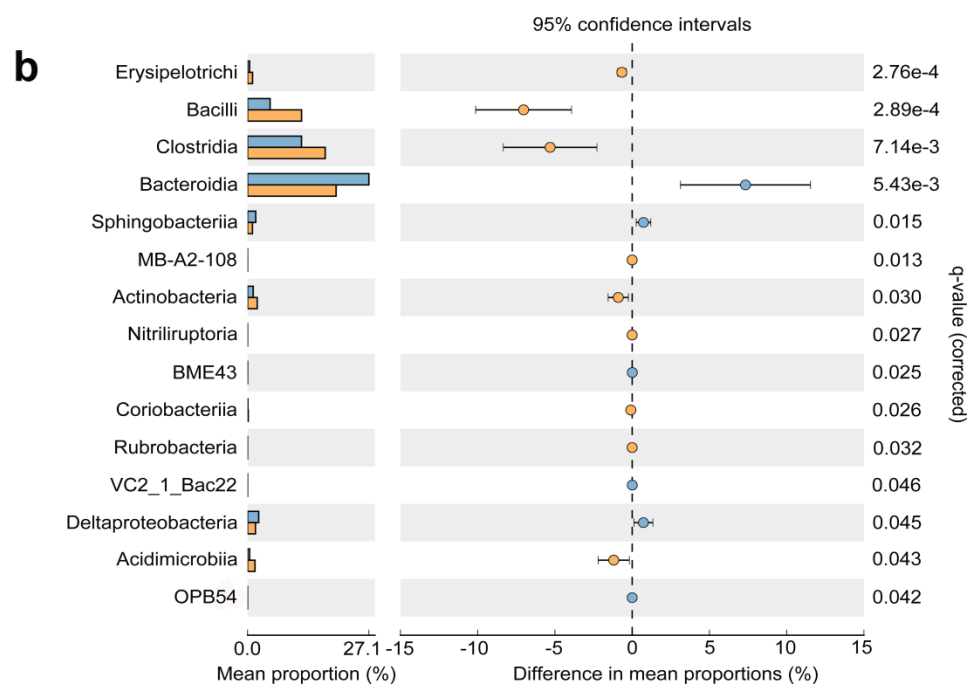

Supplement: Supplementary file 6 — Figure S2. Post-hoc plot (ANOVA, null hypothesis) indicating the mean proportion of the dominant ARG subtypes (a) and bacterial classes (b) with significant differences (adjusted P < 0.05, FDR adjusted) between summer and winter. (PDF 329 kb) [file 40168_2017_298_MOESM6_ESM.pdf]

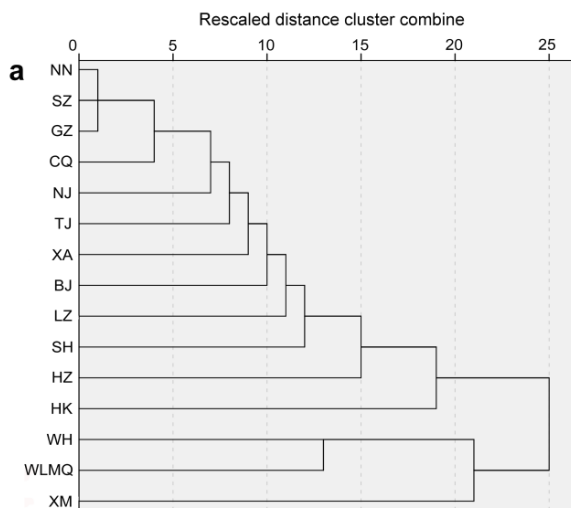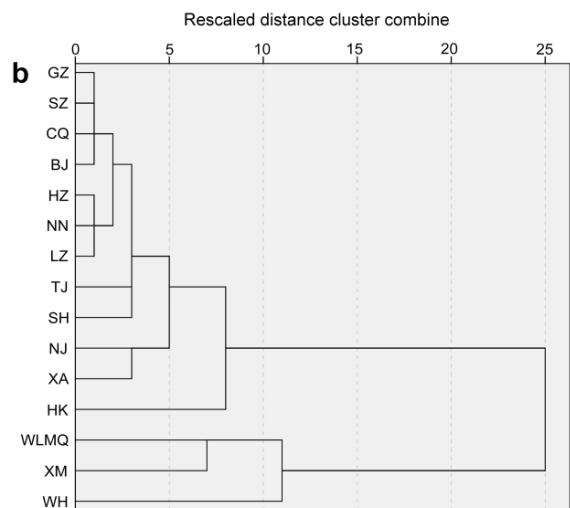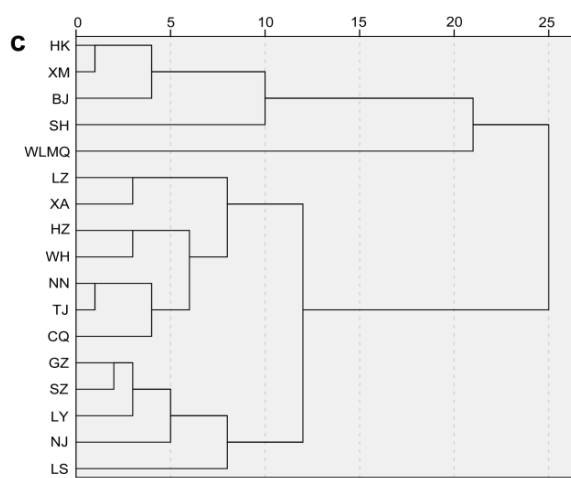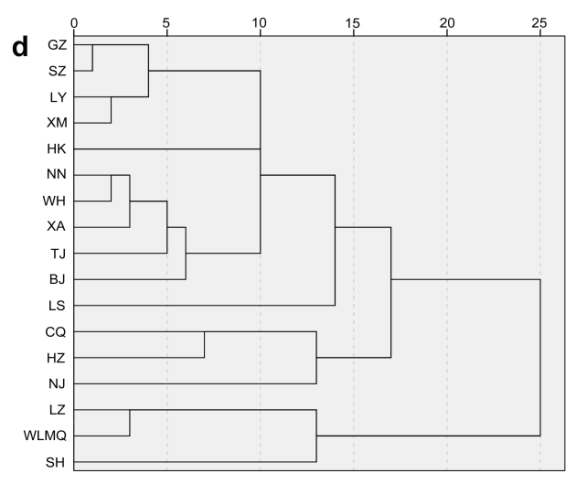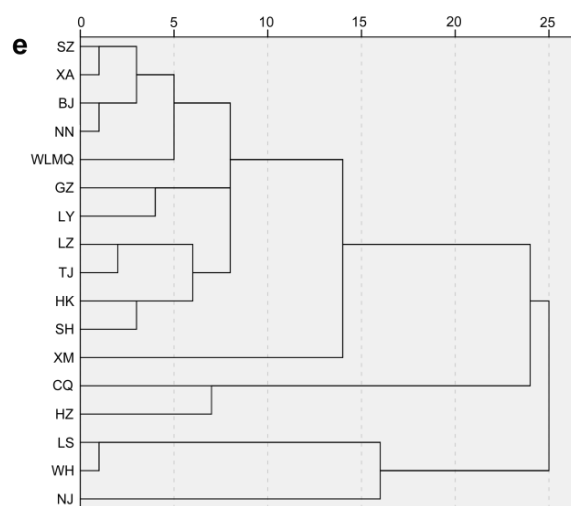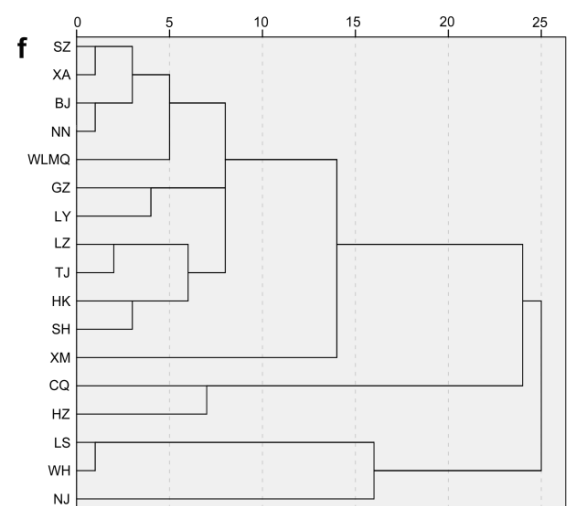

Supplement: Supplementary file 7 — Figure S3. Cluster analysis (hierarchical cluster) based on between-groups linkage method revealed that no distinct geographic clustering of resistome and bacterial community in urban sewage was observed. The Euclidian distances between two observations were measured using interval and square Euclidean distance. a All ARG subtypes. b Shared ARG subtypes. c Bacterial community. d Shared bacterial OTUs. e Human gut microbiota. f Shared human gut genera. Numbers on the top indicate rescaled distance cluster combine. (PDF 220 kb) [file 40168_2017_298_MOESM7_ESM.pdf]

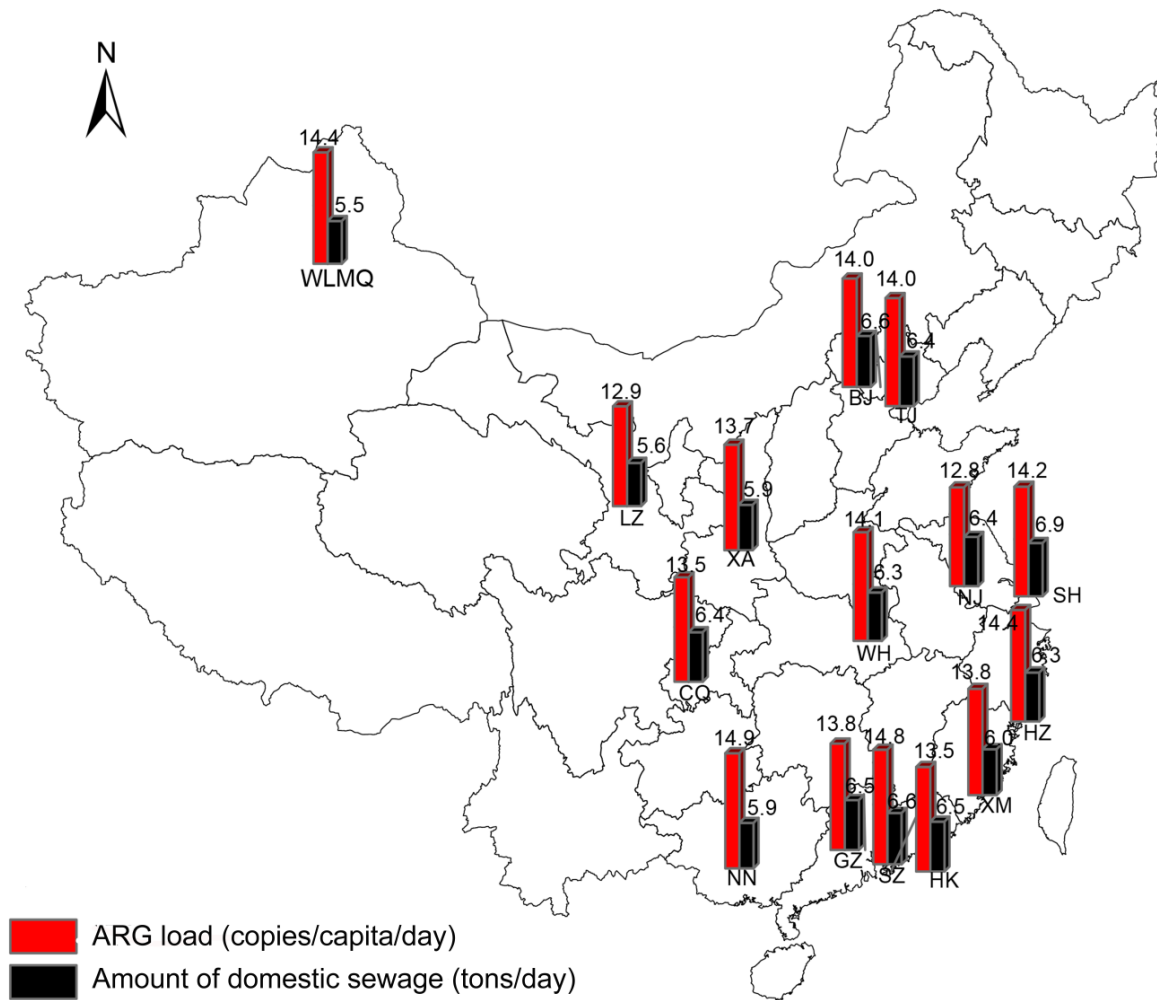

Supplement: Supplementary file 9 — Figure S4. ARG load and amount of domestic sewage discharged in major Chinese cities. The base map used is from the National Fundamental Geographic Information System of China. The red and black columns represent the average ARG load (copies/capita/day) and the total amount of domestic sewage discharged in each city (tons/day), respectively. All of the values marked in the map were standardized by log algorithm. (PDF 323 kb) [file 40168_2017_298_MOESM9_ESM.pdf]

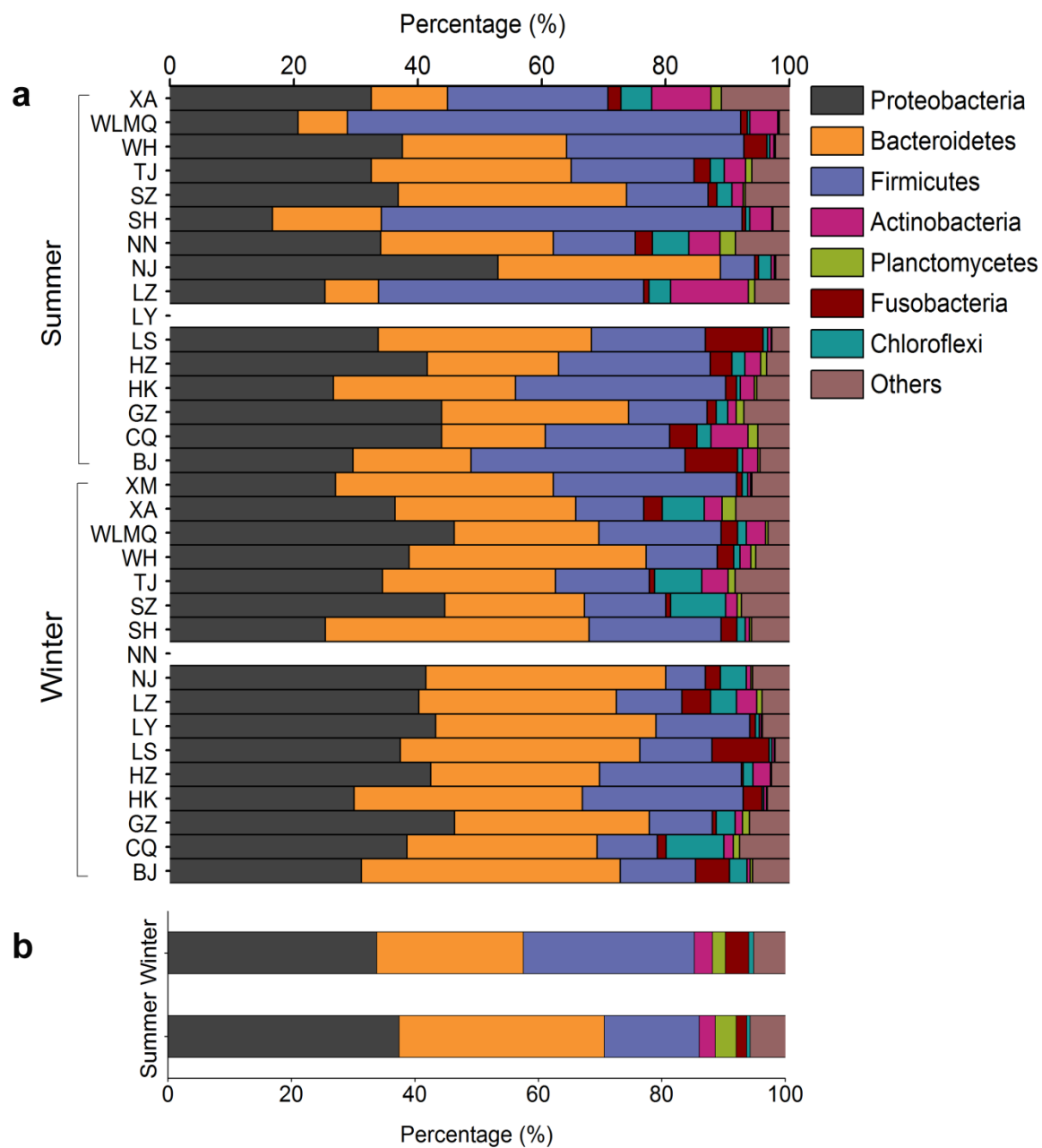

Supplement: Supplementary file 10 — Figure S5. Bacterial community compositions in sewage samples. a The relative abundance of major phyla in each city. b Comparison of the relative abundance of major phyla between summer and winter samples at the phylum level. ANOVA, * represents P < 0.05, and ** represents P < 0.01. Others refer to the phyla with the average percentage less than 2%. (PDF 230 kb) [file 40168_2017_298_MOESM10_ESM.pdf]

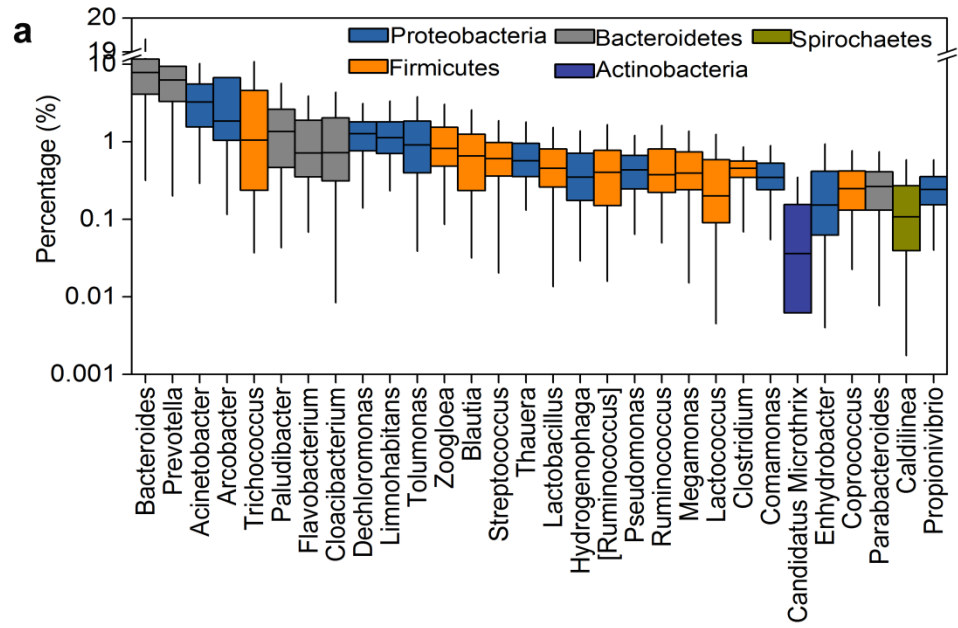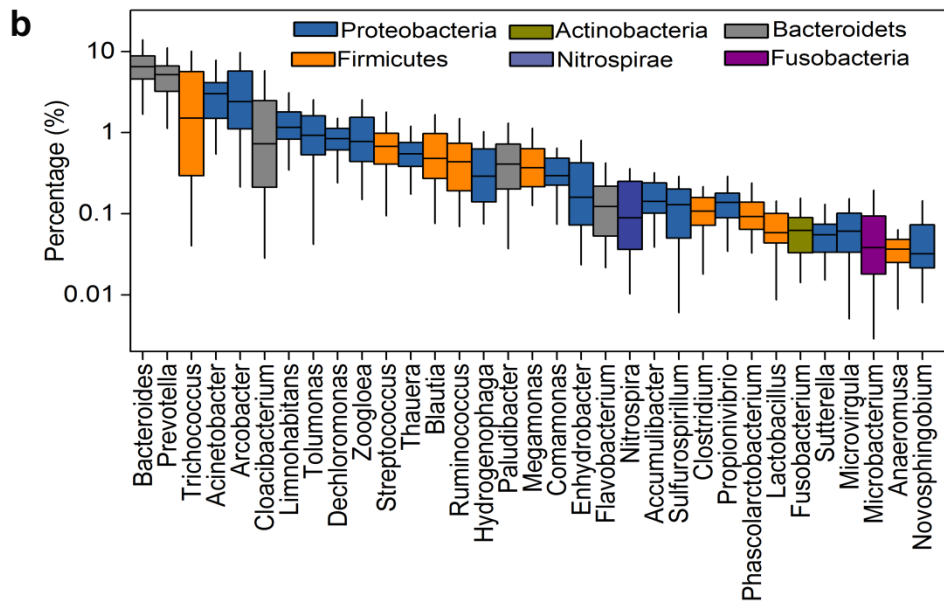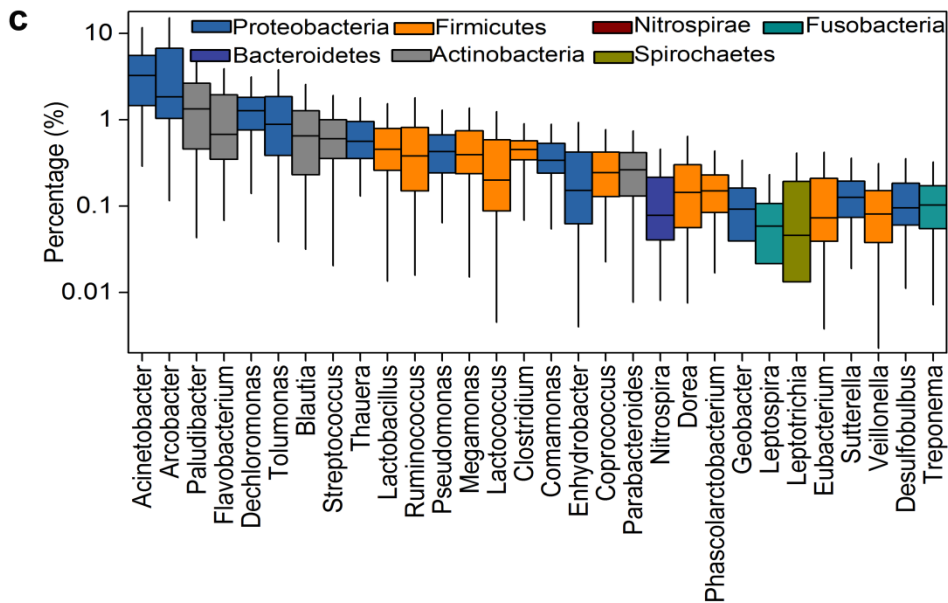

Supplement: Supplementary file 11 — Figure S6. Relative abundances of the bacterial genera in all the WWTP sewage samples. Genera are colored by their respective phylum. a The relative abundance of the top 30 abundant genera, representing as the relative abundance of each genus. b The relative abundance of the 33 shared, classified bacterial genera. c The relative abundance of top 30 human gut microbial genera. (PDF 559 kb) [file 40168_2017_298_MOESM11_ESM.pdf]

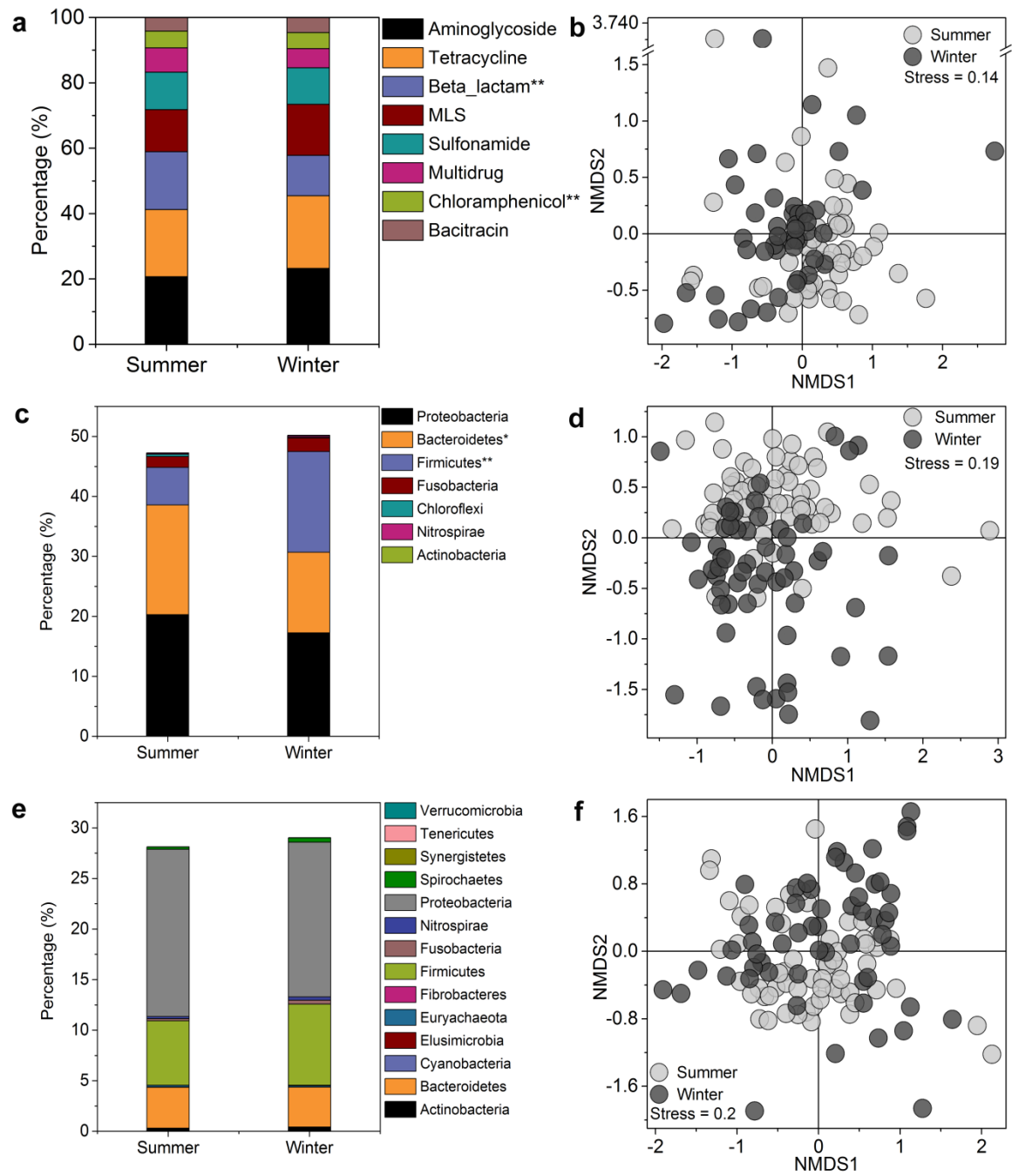

Supplement: Supplementary file 12 — Figure S7. Seasonal variation of antibiotic resistomes and microbial communities in sewage. a Percentage of the core resistomes in urban sewage samples. MLS Macrolide-Lincosamide-Streptogramin resistance. b NMDS analysis revealing the distribution pattern of the core resistomes at subtype level (Adonis tests, P < 0.01). c Relative abundance of the phyla that shared OTUs were affiliated to in WWTP sewages. d NMDS analysis of shared OTUs with seasonal change as instrumental variables based on the abundance of OTUs across 116 individual sewage samples (Adonis test, P < 0.01). e Percentage of human gut microbial phyla with the relative abundance in all of the WWTP sewages. f NMDS analysis revealing the pattern of human gut bacteria with the seasonal change (Adonis test, P < 0.01). ANOVA, * represents P < 0.05, and ** represents P < 0.01. NMDS analysis was conducted based on Bray-Curtis distance. (PDF 436 kb) [file 40168_2017_298_MOESM12_ESM.pdf]

**a**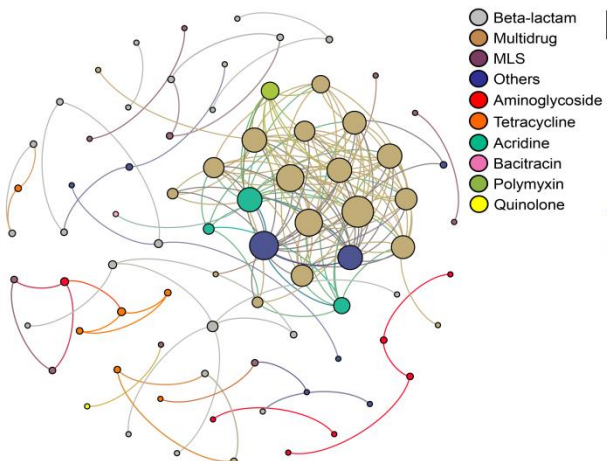**b**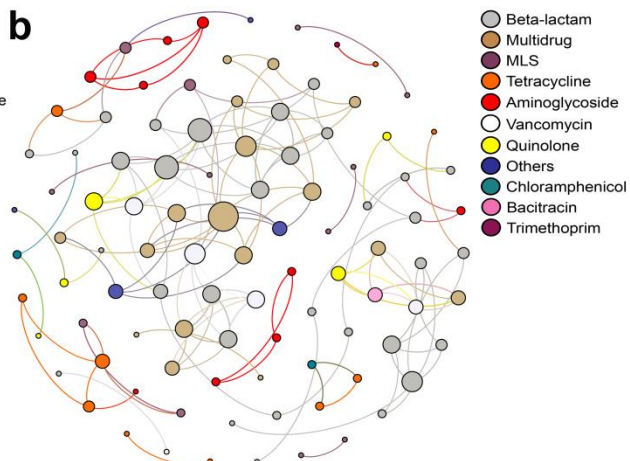**c**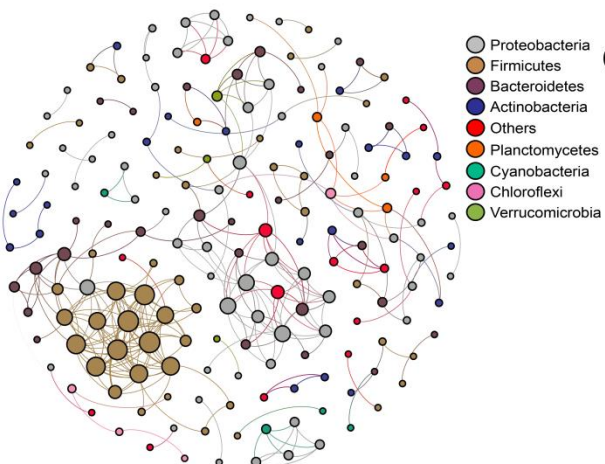**d**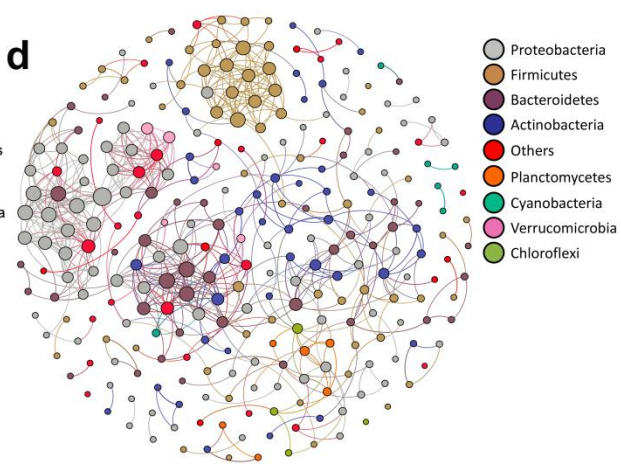

Supplement: Supplementary file 13 — Figure S8. Co-occurrence patterns of summer (a) and winter (b) antibiotic resistome and summer (c) and winter (d) microbiome in urban sewages. A connection stands for a strong (Spearman’s ρ > 0.7) and significant (P value < 0.01) correlation. Nodes indicate taxonomic affiliation at ARG subtypes (a and b) and genus level (c and d), respectively. The color of each node indicates various ARG types (a and b) and bacterial phyla (c and d). Size of the nodes was proportioned to the number of connections and the width of the edges (lines connecting the circles) was proportioned to the Spearman’s correlation coefficient. In plot a and b, MLS stands for Macrolide-Lincosamide-Streptogramin resistance and Others represent the genes coding other unclassified antibiotic resistance proteins or other functional proteins; while in plot c and d, Others refer to the unclassified phyla. (PDF 335 kb) [file 40168_2017_298_MOESM13_ESM.pdf]
